# Accidental dental displacement into the maxillary sinus during extraction maneuvers: a case series

**DOI:** 10.4317/medoral.24054

**Published:** 2020-11-28

**Authors:** Jorge Toledano-Serrabona, Jordi Cascos-Romero, Cosme Gay-Escoda

**Affiliations:** 1 DDS. Fellow of the master’s degree programme in Oral Surgery and Implantology, Faculty of Medicine and Health Sciences, University of Barcelona. Coordinator of master’s degree programme in Oral Surgery (EFHRE International University/FUCSO, Belize City, Belize). Researcher at IDIBELL (Bellvitge Biomedical Research Institute), Barcelona, Spain; 2 DDS, MS. Associate Professor of Oral Surgery. Master’s degree in Oral Surgery and Implantology, Faculty of Medicine and Health Sciences, University of Barcelona, Spain; 3 MD, DDS, MS, PhD, EBOS, OMFS. Chairman and Professor of Oral and Maxillofacial Surgery, Faculty of Medicine and Health Sciences, University of Barcelona. Director of the Master’s degree programme in Oral Surgery and Implantology (EFHRE International University/FUCSO). Coordinator/Researcher of the IDIBELL (Bellvitge Biomedical Research Institute). Head of the Oral Surgery, Implantology and Maxillofacial Surgery Department of the Teknon Medical Center, Barcelona, Spain

## Abstract

**Background:**

The aims of this study were to describe the clinical findings of patients that suffered teeth displacement into the maxillary sinus, and to report the surgical technique used to solve this complication.

**Material and Methods:**

A retrospective observational study was conducted involving patients that suffered a displacement of teeth into the maxillary sinus. Demographic and clinical data were recorded from the affected patients and a descriptive statistical analysis was made of the study variables.

**Results:**

A total of nine patients were enrolled, six males (66.7%) and three females (33.3%), with a mean age of 36.0 years (range 22-54). In five patients (55.5%) the displaced teeth remained asymptomatic; however, dental fragments were retrieved from the maxillary sinus using Caldwell-Luc technique or endoscopic approach.

**Conclusions:**

Dental displacement into the maxillary sinus during the extraction manoeuvres is an uncommon finding. Even in asymptomatic cases, these displaced teeth should be extracted in order to avoid the development of sinus pathology.

** Key words:**Maxillary sinus, dental root fragment, accidental tooth displacement, extraction, surgical complications, Caldwell-Luc.

## Introduction

Extractions of included teeth are the most common procedure in a Department of Oral and Maxillofacial Surgery. Although the overall complication rate related to this procedure is low, during or after dental extraction can occur nerve injuries, mandibular fractures, oroantral communications among other possible complications that surgeons must take into account prior the surgery (1).

Accidental displacement of tooth fragments or of complete teeth towards adjacent anatomical spaces (i.e. maxillary sinus, infratemporal fossa, buccal space, submandibular space, pterygomandibular space, and lateral pharyngeal space) is a rare event that requires a specific treatment in each case (2).

The maxillary sinus is the anatomical space located in the maxilla bone most often affected by this complication (3,4). Dental displacement towards maxillary sinus occurs more frequently during the extraction maneuvers of the maxillary first molar or the upper wisdom tooth, with an estimated prevalence of 0.6-3.8% (5,6). Among the different etiologies described in the literature, iatrogenic cases resulting from dental treatment are the most common cause of foreign bodies displaced towards this cavity, followed by trauma (7).

Some risk factors related to teeth migration to adjacent spaces have been suggested, such as the close anatomical relationship, the use of excessive and uncontrollable forces, the lack of clinical experience and the poor clinical and radiological assessment (8,9). Other aspects, such as the depth of inclusion or the teeth anatomy, including the number and shape of roots and the angulation of the third molar may increase the risk of these accidental dental displacements (10-12).

In the event of dental displacement towards the maxillary sinus, the tooth or fragment involved can lodged between the outer cortical layer and the buccal mucosa, between the floor and the mucosa of the maxillary sinus, or penetrate through the membrane of the sinus (12). An accurate diagnosis with plain radiographs or even with computed tomography images is mandatory to retrieve the foreign bodies from the maxillary sinus (11,13,14). On the other hand, according to the literature, there are three surgical approaches to remove dental fragments from the maxillary sinus; the Caldwell-Luc technique, the endoscopic procedure and the crestal route (15).

There are few studies in the literature which mention dental displacement into the maxillary sinus because its incidence is low. Thus, this report aims to describe nine clinical cases of teeth or roots displaced into the maxillary sinus, and to report the surgical technique used to solve this complication.

## Material and Methods

The local Institutional Review Board of the Teknon Medical Center (Barcelona, Spain) approved the present study (protocol number 2020/14-ODO-CMT). All participants signed consent form prior the surgery. During the course of the study, the Declaration of Helsinki were abided.

A retrospective study was carried out of nine patients that presented an accidental dental displacement into the maxillary sinus occurring during the extraction maneuvers of the posterior maxillary teeth. This complication was diagnosticated by clinical examination and plain radiographs. Computed tomography (CT) or Cone Beam CT (CBCT) were also indicated depending on the preference of the surgeons. Patients were treated in the Department of Oral Surgery and Maxillofacial Surgery at the Teknon Medical Center, Barcelona, Spain from 2000 to 2019.

The following data were collected: gender, age, medical background, type of tooth, sinus infection, and treatment performed in each case. Finally, a descriptive analysis was made using the statistical package Stata14 (StataCorp., College Station, USA).

## Results

**Figure 1:**
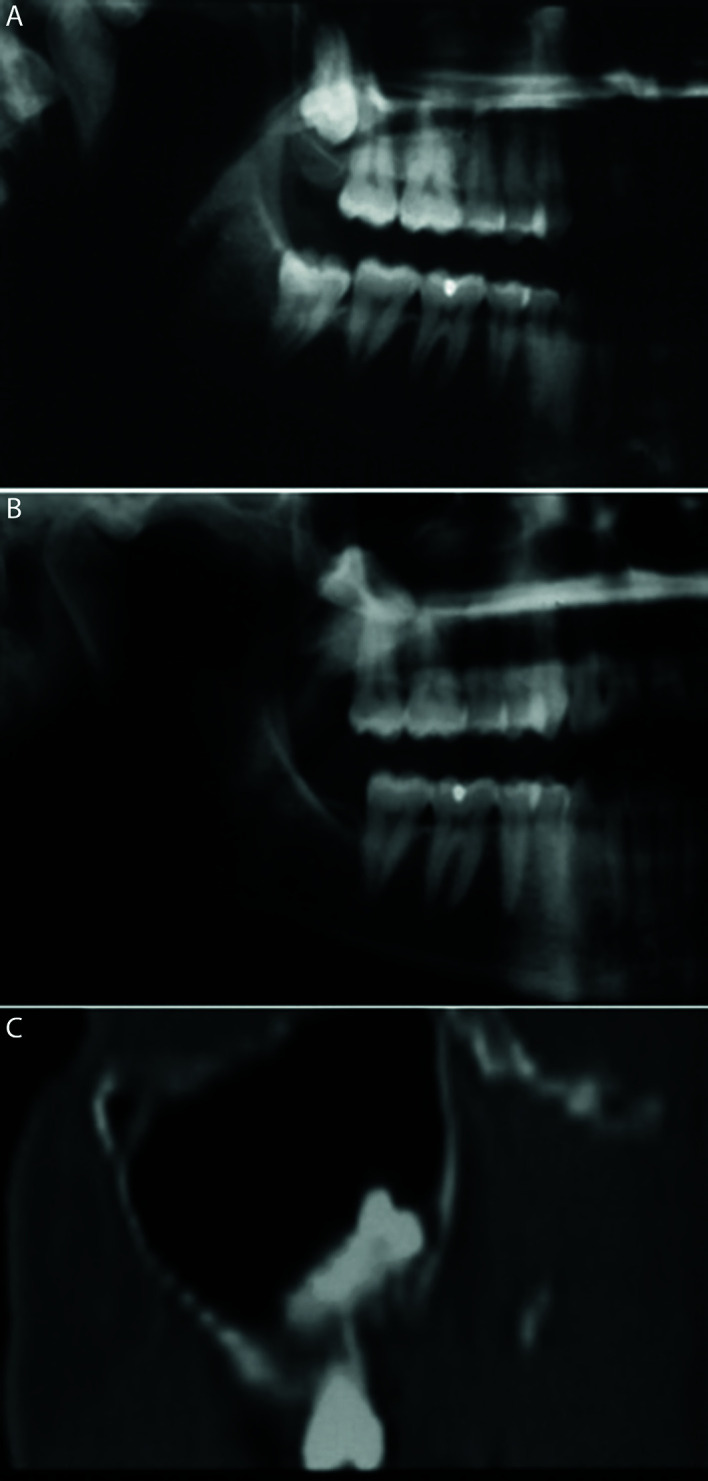
Panoramic radiograph view prior to extraction (A) and after the first attempt of removal (B). Computed tomographic view (coronal section) showing 1.8 displaced into the right maxillary sinus (C).

In total, nine patients (six males, three females) aged 22-54 (mean 36.0 years) presented root or tooth displacement into the maxillary sinus during tooth extraction maneuvers. Four patients (44.4%) had the upper wisdom teeth entirely displaced into the maxillary sinus and the rest of cases (55.6%) were palatal roots of the first or second molar. The clinical features of the sample were displayed in Table 1. All patients have a panoramic radiograph (PR) prior the removal of the upper teeth (Fig. 1).

**Figure 2:**
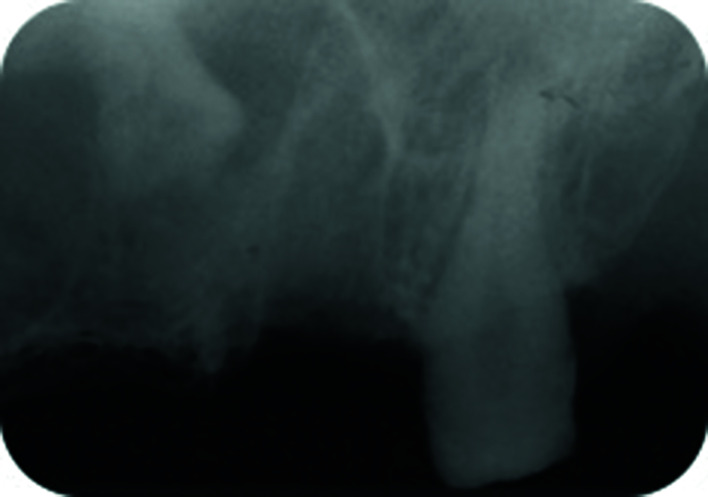
Periapical x-ray view showing the fragment of the palatal root of 2.7 (R) displaced into the left maxillary sinus.

**Figure 3:**
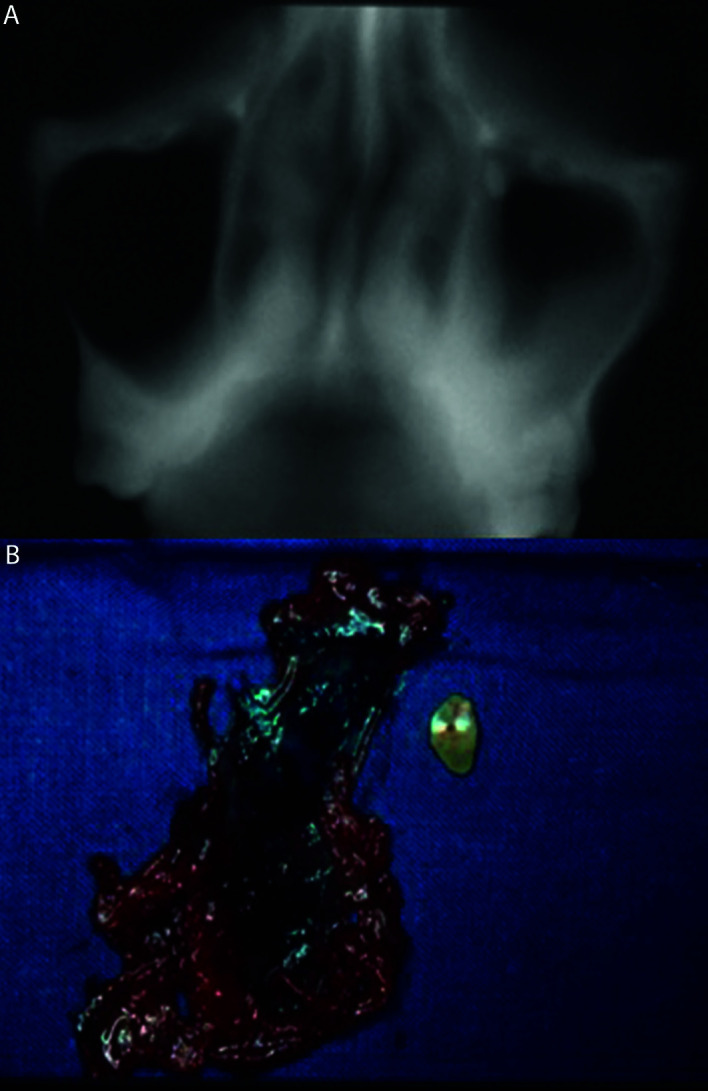
Tomographic view (coronal section) showing a fragment corresponding to the palatal root of 2.6 displaced into the left maxillary sinus (A). Root fragment and hyperplastic sinus mucosa removed during Caldwell-Luc intervention (B).

**Figure 4:**
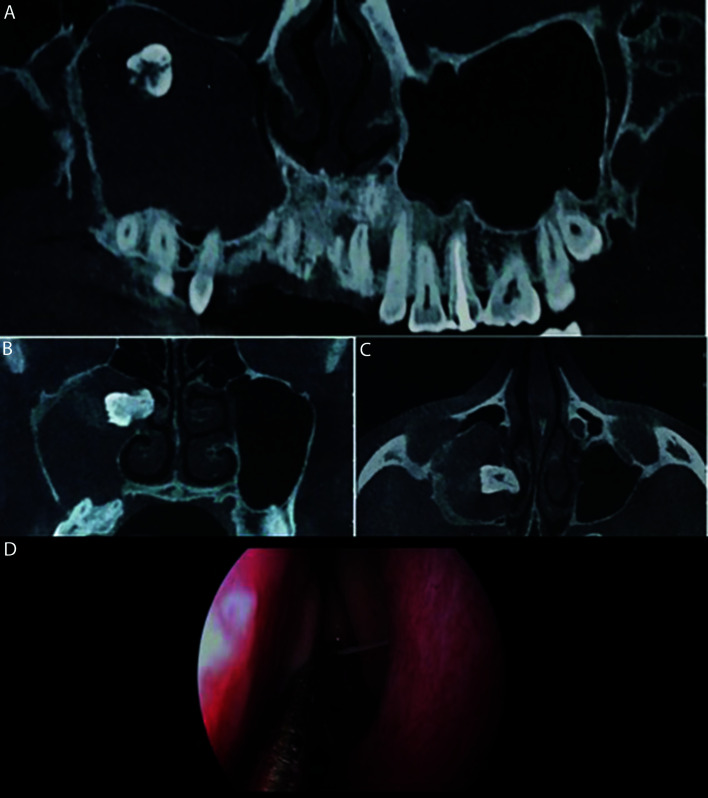
Recovery of the tooth through the nasal route (endoscopy). (A) CBCT panoramic reconstruction (B) Frontal CT scan (C) Axial CT scan. Note the tooth displaced and the signs of pathology in the right maxillary sinus. (D) Endoscopic approach view through the nasal route.

After this complication occurred, the alveolar ridge was closed by primary intention and the recovery of the teeth was done in a different appointment; after two weeks in the asymptomatic cases or after the remission of acute symptoms in the cases of sinus infection. Periapical radiographs and PRs were taken after the teeth were migrated into the sinus cavity (Fig. 1,Fig. 2), and CT or CBCT study were also performed in six cases (66.7%) in order to assess the position of the teeth and the changes in maxillary sinus mucosa (Fig. 3, Fig. 4).

Four patients (44.4%) showed an acute maxillary sinusitis infection occurred after the teeth were displaced. This condition was treated with Amoxiciline [750 g 1 Tablet every 8h for 7 days (GlaxoSmithKline, Madrid, Spain)] and analgesics (Ibuprofen [600 mg 1 Tablet every 8h for 3-5 days (Algiasdin 600; Esteve, Barcelona, Spain)] prior to the surgical approach.

In seven cases (77.8%) the Caldwell-Luc approach was performed in order to rescue the displaced teeth. Surgical procedures were made under Articaine 4% with epinephrine 1:100.000 (Ultracain, Normon; Madrid, Spain) and multimodal conscious intravenous sedation. Only in one case general anesthesia was required.

**Table 1:**
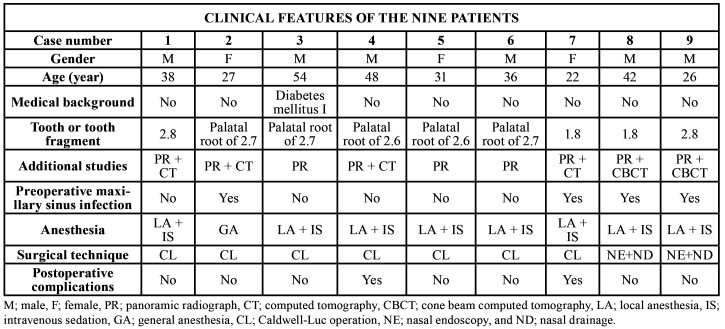
Clinical features of the sample.

Patients were placed in the supine position to fall the root into the posterior part of the maxillary sinus and supracrestal incision was placed in the alveolar ridge, with two releasing incisions in the mesial and distal aspect of the proposed area. After the flap was elevated, a sterile low-speed handpiece with a tungsten carbide drill and under profuse sterile saline irrigation was used to remove part of the bone of the lateral wall of the maxillary sinus. The sinus mucosa was perforated through the window in order to remove the displaced roots or teeth. After teeth were extracted, only hyperplastic maxillary sinus mucosa was removed (Fig. 3), leaving the healthy sinus mucosa. Finally, the mucoperiosteal flap was sutured with 3/0 silk (Silkam, Braun; Tuttlingen, Germany) by primary intention.

In the remaining two patients (22.2%) the displaced teeth were removed by transnasal endoscopy and under local anesthesia and conscious intravenous sedation (Fig. 4).

The tooth was localized and removed with the endoscope equipped with a digital video unit, and in addition, when pathological changes were detected in the sinus mucosa, it was removed with punches of the endoscope. After the operation, a nasal drainage was placed for 2 days.

The patients received an Amoxiciline [750 g 1 Tablet every 8 hours for 7 days (GlaxoSmithKline, Madrid, Spain)], and an analgesic and anti-inflamatory drugs with Ibuprofen [600 mg 1 Tablet every 8 hours for 3-5 days (Algiasdin 600; Esteve, Barcelona, Spain)] or Diclofenac [50 mg 1 Tablet every 8 hours for 3-5 days (Diclofenaco Llorens 50 mg; Llorens, Barcelona, Spain)] and Paracetamol [1g 1 Tablet every 8 hours for 3-5 days (Gelocatil; Ferrer, Barcelona, Spain)] as a rescue medication. Twenty-four hours after the procedure, the patients rinsed with a 0.12% chlorhexidine solution (Clorhexidina Lacer; Lacer, Barcelona, Spain) every 12 hours for 15 days. Postoperative instructions were explained to all patients.

No cases of intra-operative complications were found, but two patients presented a postoperative infection in the maxillary sinus that was treated with Clindamycin [300mg 1 Tablet every 6 hours for 7 days (Dalacin 300; Pfizer, Madrid, Spain)] and Budesonide [100g 2 instillations in each nares for 1 month (Budesonida Aldo-Unión; Laboratorios Aldo-Unión, Barcelona, Spain)].

## Discussion

Although foreign bodies in the maxillary sinus is an uncommon finding, dental maneuvers during the extraction of maxillary posterior teeth can cause dental displacement. Despite the low incidence of this complication, according to Hara *et al*.’s review (7), out of 407 cases of foreign bodies displaced towards maxillary sinus, 220 were tooth roots. In our sample, six males and three females presented two palatal roots of the first molar, three palatal roots of the second molar, and four wisdom teeth with fused roots displaced into the maxillary sinus.

According to previous published papers, ﻿this complication is more common in the upper wisdom tooth or the first molar, and in males patients (12). Etiology of displacement of the teeth into the maxillary sinus is multifactorial and well-known. Risk factors that can influence this complication are an inadequate clinical and radiological assessment, a lack of surgical experience with the application of excessive apical forces with the elevators, and the maxillary sinus features. Regarding the clinical and radiological evaluation, it must be pointed out the teeth anatomy, specially teeth with fused roots and conical shape, the depth of inclusion and the close relationship between the roots and the maxillary sinus (10-12). Other aspects such as the presence of a pneumatic sinus, or the existence of periapical lesions must also be duly considered before the surgery (10). Thus, antral teeth and germs with single or fused roots located in a high position and with a close relationship to the maxillary sinus are more prone to suffer this kind of complication. In this line, the four wisdom teeth displaced in our sample had fused roots with conical shape. Furthermore, all the roots involved in the present study had a close relationship with the maxillary sinus.

When dental displacement is suspected, an immediate diagnosis must be established. Clinically, it is noted the disappearance of the root fragment or the complete tooth with an oroantral communication (4). However, x-ray images are needed to verify the dental mobilization into maxillary sinus. In the vast majority of cases PR is sufficient to confirm displaced teeth but the use of CT allows to confirm the size of the dental fragment, the pathologic changes in sinus mucosa and to perform an accurate surgical plan (13,14). In our sample, the CT study was necessary in six patients.

The main complication of tooth displacement into the maxillary sinus is the appearance of maxillary sinusitis produced by the presence of an oroantral communication or the irritation produced by the displaced teeth (16). In the present sample, four patients showed clinical and radiological signs of maxillary sinusitis. The treatment of this complication comprises removal of the dental fragments, followed by the elimination of the pathological sinus mucosa adopting a CL approach or an endoscopic surgery (13,17). As Wooley and Patel (18) suggest, the untreated maxillary sinusitis may affect the other paranasal sinuses, so when signs of sinusitis appears an early treatment is mandatory to prevent future complications.

Leaving the tooth or tooth fragments is an option described in cases of accidental intraoperative displacement without signs of sinusitis and when the displaced fragments are small (18). However, the majority of the authors recommend the retrieval of these foreign bodies even in asymptomatic cases in order to prevent sinus infection and their complications (11,13).

Endoscopic retrieval and CL approach are the most described surgical methods in the literature to remove foreign bodies from the maxillary sinus cavity (13,17,19-21). In our opinion and in accordance with Huang *et al*. (11), socket via should be avoided to extract displaced elements in order to prevent the enlargement of oroantral communication. However, this approach can be indicated when there is a large defect on the alveolar ridge and there is no risk of augmentation of oroantral communication.

For a long time, CL was the treatment chosen to remove accidental displaced roots or dental implants into the maxillary sinus because is a well-described technique that allows an excellent access with a simple surgical technique (11,13). However, this approach is invasive with worse recovery and higher number of complications than minimally invasive endoscopic surgery (20,21).

In the last decades, due to the emergence of endoscope, the retrieval treatment is more conservative and well tolerated (14). Endoscopic device can be used transnasal, through a bone window in the canine fossa or through the socket. This technique allows a visualization of the maxillary sinus, a faster recovery of the patients and respects the integrity of maxillary sinus (20-22). Nevertheless, one limitation of this approach is that should be associated with local flaps in order to close oroantral communication (23,24).

## Conclusions

Within the limitations of the present study, it can be concluded that the risk of teeth displacement into the maxillary sinus is low and may be reduced by an accurate clinical and radiographic diagnosis and conducting non-traumatic dental extraction. In addition, even in asymptomatic patients, the retrieval of these dental fragments should be mandatory in order to avoid the development of sinus pathology.

## References

[1] Bailey E, Kashbour W, Shah N, Worthington HV, Renton TF, Coulthard P (2020). Surgical techniques for the removal of mandibular wisdom teeth. Cochrane Database Syst Rev.

[2] Bozkurt P, Erdem E (2017). Management of upper and lower molars that are displaced into the neighbouring spaces. Br J Oral Maxillofac Surg.

[3] Iwai T, Chikumaru H, Shibasaki M, Tohnai I (2013). Safe method of extraction to prevent a deeply-impacted maxillary third molar being displaced into the maxillary sinus. Br J Oral Maxillofac Surg.

[4] Iwai T, Matsui Y, Hirota M, Tohnai I (2012). Endoscopic removal of a maxillary third molar displaced into the maxillary sinus via the socket. J Craniofac Surg.

[5] Bouquet A, Coudert JL, Bourgeois D, Mazoyer JF, Bossard D (2004). Contributions of reformatted computed tomography and panoramic radiography in the localization of third molars relative to the maxillary sinus. Oral Surg Oral Med Oral Pathol Oral Radiol Endod.

[6] Primo BT, Stringhini DJ, da Costa DJ, Rebellato NLB, Scariot R (2016). Delayed removal of maxillary third molar displaced into the maxillary sinus. Stomatologija.

[7] Hara Y, Shiratsuchi H, Tamagawa T, Koshi R, Miya C, Nagasaki M (2018). A large-scale study of treatment methods for foreign bodies in the maxillary sinus. J Oral Sci.

[8] Aznar-Arasa L, Figueiredo R, Gay-Escoda C (2012). Iatrogenic displacement of lower third molar roots into the sublingual space: Report of 6 cases. J Oral Maxillofac Surg.

[9] Gay-Escoda C, Berini-Aytés L, Piñera-Penalva M (1993). Accidental displacement of a lower third molar. Report of a case in the lateral cervical position. Oral Surg Oral Med Oral Pathol.

[10] Sencimen M, Gülses A, Secer S, Zerener T, Özarslantürk S (2017). Delayed retrieval of a displaced maxillary third molar from infratemporal space via trans-sinusoidal approach: A case report and the review of the literature. Oral Maxillofac Surg.

[11] Huang IY, Chen CM, Chuang FH (2011). Caldwell-Luc procedure for retrieval of displaced root in the maxillary sinus. Oral Surg Oral Med Oral Pathol Oral Radiol Endod.

[12] Asmael HM (2018). The modified Caldwell-Luc approach in retrieval of accidentally displaced root into the maxillary sinus. J Craniofac Surg.

[13] Ridaura-Ruiz L, Figueiredo R, Guinot-Moya R, Piñera-Penalva M, Sanchez-Garcés MA, Valmaseda-Castellón E (2009). Accidental displacement of dental implants into the maxillary sinus: A report of nine cases. Clin Implant Dent Relat Res.

[14] Sgaramella N, Tartaro G, D'Amato S, Santagata M, Colella G (2016). Displacement of dental implants into the maxillary sinus: A retrospective study of twenty-one patients. Clin Implant Dent Relat Res.

[15] Wang H, Yang CY, Li Z (2018). Traumatic displacement of teeth into maxillary sinus and the retrieval assisted by computer-assisted navigation: A case report. Medicine (Baltimore).

[16] Chang PH, Chen YW, Huang CC, Fu CH, Huang CC, Lee TJ (2020). Removal of displaced dental implants in the maxillary sinus using endoscopic approaches. Ear Nose Throat J.

[17] Gao QM, Yang C, Zheng LY, Hu YK (2016). Removal of long-term broken roots displaced into the maxillary sinus by endoscopic assistant. J Craniofac Surg.

[18] Woolley EJ, Patel M (1997). Subdural empyema resulting from displacement of a root into the maxillary antrum. Br Dent J.

[19] Amin N, Walker A, Alobid I, Anari S, Bast F, Bhalla RK (2020). Defining appropriateness criteria for endoscopic sinus surgery in the management of adult dental implant patients with incidental maxillary sinus findings on cone beam computed tomography. Clin Otolaryngol.

[20] Safadi A, Ungar OJ, Oz I, Koren I, Abergel A, Kleinman S (2020). Endoscopic sinus surgery for dental implant displacement into the maxillary sinus- A retrospective clinical study. Int J Oral Maxillofac Surg.

[21] An JH, Park SH, Han JJ, Jung S, Kook MS, Park HJ (2017). Treatment of dental implant displacement into the maxillary sinus. Maxillofac Plast Reconstr Surg.

[22] Chandrasena F, Singh A, Visavadia BG (2010). Removal of a root from the maxillary sinus using functional endoscopic sinus surgery. Br J Oral Maxillofac Surg.

[23] Matti E, Emanuelli E, Pusateri A, Muniz CCS, Pagella F (2013). Transnasal endoscopic removal of dental implants from the maxillary sinus. Int J Oral Maxillofac Implants.

[24] Lim D, Parumo R, Chai MB, Shanmuganathan J (2017). Transnasal endoscopy removal of dislodged dental implant: A case report. J Oral Implantol.

